# Ophthalmic surgical simulator: an effective and safe approach for trainees in vitreoretinal surgery

**DOI:** 10.1007/s10792-025-03851-5

**Published:** 2025-12-12

**Authors:** Federico Giannuzzi, Lorenzo Hu, Matteo Mario Carlà, Valentina Cestrone, Sofia Marcelli, Umberto De Vico, Alfonso Savastano, Emanuele Crincoli, Maria Cristina Savastano, Stanislao Rizzo

**Affiliations:** 1 Ophthalmology Unit, Fondazione Policlinico Universitario A. Gemelli, IRCCS, 00168 Largo A. Gemelli, 8, Rome, Italy; 2 Catholic University “Sacro Cuore”, Rome, Italy; 3 Libera Università Mediterranea Degennaro, Casamassima, Bari Italy; 4 Ospedale Generale Regionale “F. Miulli”, Acquaviva delle Fonti, Bari Italy

**Keywords:** EyeSi, Surgical simulator, Vitreoretinal surgery, Stereopsis, Fusional amplitude

## Abstract

**Purpose:**

This study aims to evaluate the safety and efficacy of the EyeSi Surgical Simulator (VRmagic, Mannheim, Germany) in enhancing surgical proficiency among trainees performing vitreoretinal procedures.

**Methods:**

Trainees underwent a comprehensive ophthalmic and orthoptic examination at baseline (T0) and 30 min after completing the simulation session (T1), which included the cover-uncover test, ocular motility assessment, fusional amplitudes, convergence measurements, and stereopsis evaluation using the TNO stereotest. A questionnaire evaluated 18 symptoms for frequency, severity, and bothersomeness. Performance scores from the first and final 15 min on the same module were compared.

**Results:**

Orthoptic examinations were conducted before and 30 min after virtual reality (VR) simulator use on 34 trainees. Far fusional amplitude increased significantly both without (19.4 ± 8.8 Δ vs. 23.9 ± 9.7 Δ, *p* = 0.003) and with striated lenses (18.2 ± 10.4 Δ vs 20.5 ± 9.1 Δ, *p* = 0.02). Near fusional amplitude remained unchanged, regardless of striated glasses (*p* = 0.52). At baseline, TNO score was 60 arcsec in 82% of trainees and 120 arcsec in 18%. After VR simulation, 52% of eyes scored 60 arcsec, 41% scored 120 arcsec, and 7% scored 240 arcsec (*p* = 0.02). Ocular discomfort was reported by 32% of trainees, with symptoms rated as mild. All trainees’ performance score improved from initial to the final simulation session.

**Conclusions:**

The EyeSi surgical simulator enhances surgical proficiency in vitreoretinal procedures with a minor, short-term reduction on stereopsis and a low incidence of mild discomfort.

## Introduction

Ophthalmic surgical training presents considerable challenges, requiring high levels of precision and skill, typically acquired through years of guided clinical practice. Virtual reality (VR) technology has opened new opportunities for surgical training, offering new methods for skill development, particularly in ophthalmology. Among the most advanced technologies in this field is the EyeSi Surgical Simulator (Vrmagic, Mannheim, Germany), which offers an immersive and risk-free environment where trainees can safely develop and refine their surgical skills, especially in cataract and vitreoretinal surgeries. [1–4] The EyeSi simulator mimics microsurgical environments, utilizing tactile feedback, high-definition visuals, and performance metrics to enhance training. Its design includes a stereoscopic display, a simulated eye model, and foot pedal controls, allowing to reproduce real-life surgical procedures and enhancing essential skills such as depth perception and fine motor coordination. The platform supports interchangeable interfaces for both vitreoretinal and cataract surgery training. [5]

Over recent years, VR-based simulators like EyeSi have been applied to various stages of surgical training and are now a key component in many residency programs. [6, 7]

In cataract surgery, use of the EyeSi simulator has been associated with lower complication rates, showing its potential to improve performance and patient safety when integrated into structured training programs. [8] For vitreoretinal surgery, which requires advanced skills in terms of depth perception, fine motor control, and spatial awareness, simulators like EyeSi provides valuable opportunities for objective feedback and skill acquisition in a controlled environment. [1]

Despite its educational benefits, concerns have been raised about potential visual fatigue and discomfort associated with prolonged VR use. Similar to 3D imaging technologies, VR simulators may cause symptoms like eye strain and blurred vision, due to the mismatch between accommodation and convergence demands. [9, 10] Specifically, EyeSi’s stereoscopic display has been occasionally reported to cause visual discomfort among users. [5] While previous studies have demonstrated the EyeSi’s ability to improve surgical performance, limited evidence exists on its effects on trainees’ visual function or long-term user experience, particularly for vitreoretinal surgery.

This study aims to evaluate the safety and effectiveness of the EyeSi Surgical Simulator in vitreoretinal surgery training. By comparing trainees’ performance in critical visual and motor tasks before and after VR simulation, we aim to provide evidence on EyeSi’s role in enhancing both technical proficiency and user experience. Moreover, this research evaluates the simulator’s influence on functional ophthalmological parameters, such as stereopsis and fusional amplitude, to assess any unintended effects of prolonged simulator use on visual function. This research contributes to the growing evidence supporting VR simulation as a key tool in modern ophthalmic training.

## Materials and methods

This prospective, single-center study evaluated the impact of the EyeSi virtual reality surgical simulator on vitreoretinal surgery training at Fondazione Policlinico Universitario A. Gemelli, IRCCS, Rome, Italy. The study was conducted in accordance with the tenets of the Declaration of Helsinki and was approved by the Institutional Review Board of the Catholic University of Rome (protocol code: 3735). Signed informed consent was obtained from each enrolled trainee.

The study involved a single group of 34 ophthalmology trainees, each of whom participated in a 90-min training session on the EyeSi simulator, specifically focused on vitreoretinal procedures. All participants were senior ophthalmology residents from multiple universities, each with basic cataract surgery proficiency; a minimum of 20 cataract procedures performed as the primary surgeon was required for inclusion. Individuals with eye conditions that cause permanent visual impairments were excluded from participation.

### Performance assessment

The vitreoretinal training module of the EyeSi simulator used in this study is designed to replicate the complexities of posterior segment surgery, allowing trainees to perform highly specialized tasks such as membrane peeling, retinal detachment repair, and laser photocoagulation. The simulator incorporates advanced tactile feedback and visual immersion through a stereoscopic display and a Binocular Indirect Ophthalmomicroscope (BIOM)-like optical system, enabling realistic simulation of intraocular procedures. Each training module provides graded difficulty levels, starting with abstract exercises like anti-tremor and bimanual coordination tasks, progressing to detailed surgical steps such as fluidics control and precise vitreous removal. Trainees completed Module-B, Level 2 on Navigation and Instruments Laser Coagulation of the vitreoretinal training course. The task required identifying four peripheral retinal holes, often necessitating wide-field viewing and simulated scleral indentation, and surrounding each retinal hole with at least two contiguous rows of laser spots while avoiding the macula, optic disc, and retinal vessels. The simulator reports a performance score (maximum points 100) based on five criteria: target achievement, efficiency, instrument handling, microscope handling and tissue treatment. To evaluate performance progression during the simulation, task scores from the first 15 min (pre-training) and the final 15 min (post-training) on the same module were extracted and compared.

### Orthoptic assessment

All participants underwent comprehensive ophthalmological and orthoptic evaluations at baseline (T0) and 30 min after completing the simulation session (T1). The assessment included cover-uncover test, ocular motility examination, measurement of fusional amplitudes at near and distance (with and without Bagolini striated lenses), evaluation of convergence, and assessment of stereopsis using the TNO stereotest.

### Safety assessment

The trainees received a questionnaire (Table 1) listing 18 specific symptoms and were asked to rate any symptoms they experienced 30 min after completing 90 min of vitreoretinal surgery simulation (T1). Each symptom was evaluated based on its frequency, severity, and bothersomeness. Participants rated each parameter on a three-point scale (0: none, 1: mild, 2: severe). The maximum score was 108. Scores below 54 were classified as “no discomfort”, scores between 54 and 81 as “minimal discomfort”, and scores ranging from 81 to 108 as “significant discomfort”. [11]

**Table 1:** Questionnaire. Participants rated each parameter on a three-point scale (0: none, 1: mild, 2: severe)

No	Symptoms	Frequency			Severity			Bothersome-ness		
1	Light bothering	0	1	2	0	1	2	0	1	2
2	Foggy vision	0	1	2	0	1	2	0	1	2
3	Blurred vision	0	1	2	0	1	2	0	1	2
4	Double images	0	1	2	0	1	2	0	1	2
5	Haloes	0	1	2	0	1	2	0	1	2
6	Excessive blinking	0	1	2	0	1	2	0	1	2
7	Foreign body sensation	0	1	2	0	1	2	0	1	2
8	Lacrimation	0	1	2	0	1	2	0	1	2
9	Itchy eye	0	1	2	0	1	2	0	1	2
10	Burning eye	0	1	2	0	1	2	0	1	2
11	Red eye	0	1	2	0	1	2	0	1	2
12	Orbital pain	0	1	2	0	1	2	0	1	2
13	Ocular heaviness	0	1	2	0	1	2	0	1	2
14	Headache	0	1	2	0	1	2	0	1	2
15	Sickness	0	1	2	0	1	2	0	1	2
16	Fatigue	0	1	2	0	1	2	0	1	2
17	Dizziness	0	1	2	0	1	2	0	1	2
18	Difficulty in digesting	0	1	2	0	1	2	0	1	2

### Statistical analysis

Data were analyzed using GraphPad PRISM Software (Version 9.0; GraphPad, La Jolla, CA, USA). The Shapiro–Wilk test was used to assess normality, with a *p*-value greater than 0.05 indicating a normal distribution. Paired t-tests were used for comparisons between pre- and post-simulation data for normally distributed variables, while the Wilcoxon signed-rank test was applied for non-parametric variables. Confidence intervals were calculated for all pairwise comparisons. To evaluate differences between groups for pre- and post-simulation results, a two-way ANOVA was employed. For categorical variables, contingency analyses were performed using Chi-square and Fisher’s exact tests. Additionally, correlation analyses were conducted to explore the relationships among continuous variables within the dataset. Results are presented as mean ± standard deviation, with statistical significance set at p < 0.05.

## Results

A total of 34 trainees (68 eyes), were included in the analysis. Among them, 25 (73.5%) were male and 9 (26.5%) were female. The mean age at the time of evaluation was 27.4 ± 4.71 years (range from 24 to 31 years). Best corrected visual acuity (BCVA), assessed using the Snellen chart, was 20/20 in all 68 eyes (100%). Of the participants, 22 (64.7%) reported using corrective lenses, including 82.4% who wore eyeglasses and 17.6% who used contact lenses. Demographic characteristics of trainees are shown in Table 1.

### Performance assessment

**Fig. 1:**
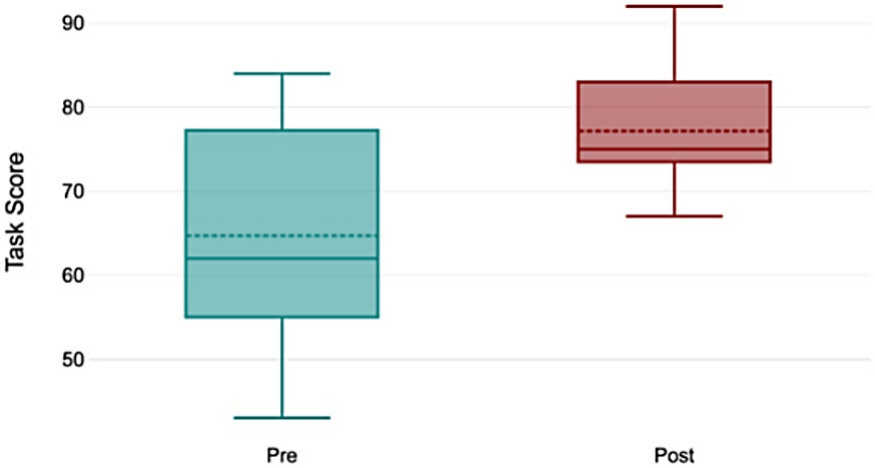
Distribution of task scores pre- and post-training. Post-training scores show significant improvement (*p* = 0.02) with reduced variability

The performance results from the pre- and post-training assessments are summarized in Fig. 1. All trainees demonstrated an improvement in their overall task scores following training with the ophthalmic surgical simulator. The mean task score increased significantly from 64.71 ± 12.57 in the pre-training session to 77.15 ± 7.05 in the post-training session (*p* = 0.02).

### Orthoptic assessment

All trainees’ fusional and convergence eye movements were within normal limits and showed no changes at T1. The mean cover test value performed at distance (6 m) was 0 Δ, indicating the absence of strabismus, both at baseline and at T1 for all participants (Table 2).

**Table 2:** Demographic characteristics of trainees

Variables	Population
Male; No (%)	25 (73.5%)
Female; No (%)	9 (26.5%)
Mean age at evaluation; years	27.4 ± 4.71 years (range, 24–31 years)
Wearing eyeglasses or contact lenses; No (%)	22 (64.7%)

**Fig. 2:**
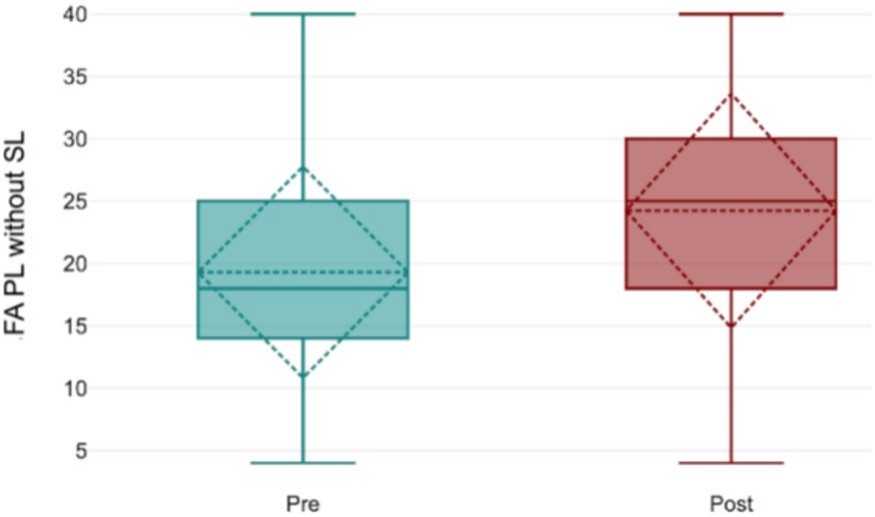
Far fusional amplitude without striated lenses. Significant increase observed post-training (*p* = 0.003)

**Fig. 3:**
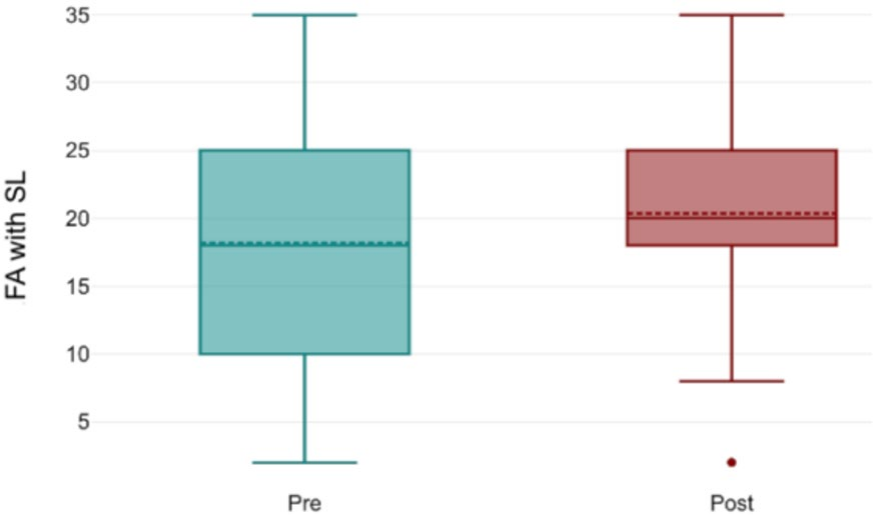
Far fusional amplitude with striated lenses. Post-training scores indicate a significant improvement (*p* = 0.02)

**Fig. 4:**
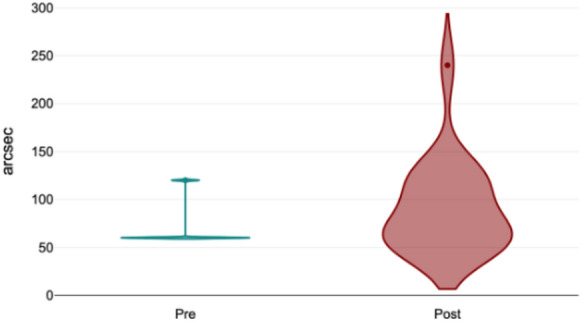
Stereopsis (TNO test) before and after training. Increased post-training variability with a shift toward higher values (*p* = 0.02)

Far fusional amplitude without striated lenses significantly increased from 19.4 ± 8.8 Δ (T0) to 23.9 ± 9.7 Δ (T1; *p* = 0.003, Fig. 2). With striated lenses, far fusional amplitude also increased significantly, from 18.2 ± 10.4 Δ to 20.5 ± 9.1 Δ (*p* = 0.02, Fig. 3). However, near fusional amplitude did not show any significant change, either with or without striated lenses (*p* = 0.52). For stereopsis, measured using the TNO test, 82% of trainees had a baseline score of 60 arcsec and 18% had 120 arcsec. After using the simulator, only 52% retained a score of 60 arcsec, while 41% shifted to 120 arcsec, and 7% to 240 arcsec (*p* = 0.02, Fig. 4). A two-way ANOVA analysis comparing scores from the initial and final training sessions showed a statistically significant increase across sessions (*p* < 0.0001). Notably, discomfort was reported by only 15% of participants, indicating a low incidence of adverse effects associated with simulator use.

### Safety assessment

All participants reported a score less than 54 on the questionnaire. The safety assessment revealed no reports of severe symptoms or bothersome effects rated at level 2. Mild adverse effects were reported by a small proportion of trainees, specifically ocular heaviness in 6 individuals (17.6%) and excessive blinking in 5 (14.7%).

## Discussion

The primary objective of the EyeSi simulator is to improve surgical training by providing a controlled environment where trainees can practice and refine their skills in complex procedures like vitreoretinal surgery. Carr et al. have demonstrated the simulator's construct validity, highlighting its effectiveness in improving surgical technique and technical errors among trainees. [12–14] By replicating realistic surgical scenarios, the EyeSi allows trainees to achieve better hand–eye coordination and depth perception, both of which are critical for successful vitreoretinal surgery. [15, 16]

The vitreoretinal module of the EyeSi simulator is a valuable adjunct for advancing surgical proficiency, offering trainees the opportunity to practice essential tasks such as precise membrane manipulation and fluidics control. Our findings of improved far fusional amplitudes and enhanced hand–eye coordination align with the module's design focus, which emphasizes the development of fine motor control and depth perception critical for posterior segment surgeries. The graded structure of the module ensures a progressive learning curve, starting with fundamental skills and advancing to complex procedures, allowing trainees to gain confidence progressively. Rasmussen et al. highlighted that VR-based training improves visuomotor control, an essential component for high-level motor skills like membrane peeling in the posterior segment. [1]

The orthoptic assessment showed a significant increase in far fusional amplitude, no change in near fusional amplitude, and a transient reduction in stereo-acuity on the TNO test. Regarding the improvement in far fusional amplitude, this may be associated with the demands placed by the VR simulator. The EyeSi system requires precise and dynamic control of eye movements, involving repeated convergence and divergence movements to navigate the virtual reality environment and perform tasks. This active engagement can function as a form of visual exercise. [17]

Stereopsis, the brain’s ability to combine disparate images into a single image with depth, is highly dependent on precise vergence. The EyeSi, like other stereoscopic/VR systems, presents disparity cues that stimulate vergence, while the accommodative demand remains fixed at the optical plane of the display. This results in a vergence-accommodation conflict (VAC), where eyes must converge to perceive virtual depth, but accommodation remains fixed at the display’s focal plane. VR-based orthoptic training has shown to improve stereoacuity in individuals with pre-existing binocular vision deficits, such as amblyopic eyes or intermittent exotropia, studies on healthy individuals with normal baseline stereopsis have reported no significant improvement or even transient worsening after exposure [17–19]. In our cohort, the shift towards higher TNO stereoacuity values, indicating poorer stereoacuity, could reflect transient visual fatigue due to the demands of the VR environment and the VAC.

Further research with larger samples and different stereopsis assessments could clarify how VR simulators interact with different aspects of visual functions over time. Moreover, the substantial cost of the EyeSi simulator remains a key limitation, potentially limiting its accessibility in smaller or resource-limited training programs. [20]

The low incidence of reported discomfort among participants, such as ocular fatigue and increased blinking, suggests that EyeSi is a well-tolerated training tool. This supports its growing integration into ophthalmology training programs as an effective, low-risk addition to traditional surgical education. [4, 7] As with any simulation tool, however, further studies examining long-term outcomes and optimal training duration are suggested to refine its application in specialized fields like vitreoretinal surgery.

## Conclusion

The EyeSi Surgical Simulator appears to be an effective and well-tolerated tool for vitreoretinal surgery training, with observed improvements in far fusional amplitudes and hand–eye coordination. Future research should explore its long-term outcomes and how its use can be optimized to enhance its role in ophthalmic education.

## Data Availability

The data that support the findings of this study are available from the corresponding author, Lorenzo Hu upon reasonable request.
